# Implementation of a controlled DCD program in Western Austria – key considerations and insights

**DOI:** 10.3389/ti.2026.16147

**Published:** 2026-06-12

**Authors:** Felix J. Krendl, Silvia Oberparleiter, Florian Ponholzer, Franka Messner, Stefan Scheidl, Manuel Maglione, Annemarie Weissenbacher, Thomas Resch, Rupert Oberhuber, Lukas Stastny, Julia Dumfarth, Clemens Seelmaier, Wolfgang List, Ronny Beer, Benno Cardini, Stefan Schneeberger, Stephan Eschertzhuber

**Affiliations:** 1 Department of Visceral, Transplant and Thoracic Surgery, Center for Operative Medicine, Medical University of Innsbruck, Innsbruck, Austria; 2 Department of Cardiac Surgery, Medical University of Innsbruck, Innsbruck, Austria; 3 Department of Cardiology and Intensive Care Medicine, Paracelsus Medical University of Salzburg, Salzburg, Austria; 4 Department of Anesthesia and Intensive Care Medicine, Hospital Feldkirch, Feldkirch, Austria; 5 Department of Neurology, Medical University of Innsbruck, Innsbruck, Austria; 6 Department of Anesthesia and Intensive Care Medicine, Hospital Hall, Hall, Austria

**Keywords:** DCD (donation after circulatory death), donation, NRP, organ procurement, program development

## Abstract

Controlled donation after circulatory death (cDCD) offers an opportunity to expand the deceased donor pool, yet implementation remains limited in many countries. We conducted a retrospective single-center analysis of all cDCD donors (Maastricht category III) referred to the Transplant Center at the Medical University of Innsbruck between January 1, 2018, and December 31, 2024. Donor characteristics, ischemia times, organ utilization, and program-level trends were analyzed. In addition, key steps and protocols essential for establishing a cDCD program were systematically evaluated. Of 56 referred cDCD donors, 53 (94.6%) proceeded to organ recovery (i.e., actual donors), and 42 (75.0%) resulted in the transplantation of at least one organ (i.e., utilized donors). Utilized donors had significantly lower BMI than non-utilized donors (25 vs. 31 kg/m^2^, p = 0.003). The median functional warm ischemia time was 26 min (IQR 23–28). The mean number of transplanted organs per donor was 2.06. Organ utilization rates were highest for kidneys (60.4%). Nationwide DCD activity increased from 3% to 18% following the implementation of a structured cDCD program in Western Austria. In summary, we have outlined steps and protocols required to successfully implement a cDCD program, resulting in high utilization rates and a measurable impact on national cDCD activity.

## Graphical Abstract

**Figure:**
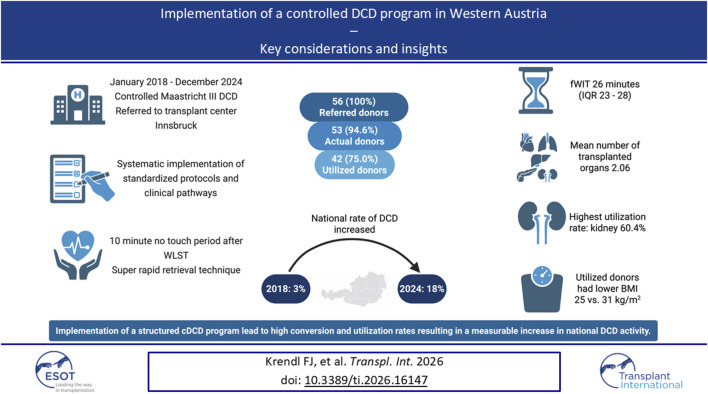
Created in BioRender. Krendl, F. J. (2026) https://BioRender.com/do87s66.

## Introduction

Before the Harvard brain death criteria had been published in 1968 [1], all deceased donors were declared dead using circulatory arrest criteria [2]. After the legal framework for the diagnosis of brain death according to neurologic criteria had been established, most countries almost exclusively relied on BD (brain-dead) donors [2]. However, due to the increasing shortage of suitable donor organs, transplant organizations have explored different strategies to expand the donor pool. One such strategy is the implementation of donation after circulatory determination of death (DCD) [3].

Outcomes following transplantation of DCD organs have historically been inferior compared to transplantation of DBD organs [4, 5], prompting many centers to be cautious in accepting DCD organs for transplantation. Yet, with improved preservation strategies entering routine clinical practice [6–8] and better donor-recipient matching [9], outcomes following DCD transplantations are beginning to equal those of DBD transplantation [10–14]. Furthermore, the utilization of DCD organs has led to decreased waitlist times and waitlist mortality rates [15–18]. Thus, in an era of continued organ supply-demand mismatch, increased recovery and utilization of DCD organs should be encouraged [16]. Globally, DCD accounts for approximately 20% of all deceased donors used for transplantation [19]. In Europe and even within the Eurotransplant network, the DCD landscape is heterogenous. While in Germany DCD is currently prohibited by law, the Netherlands and Belgium run very successful DCD programs with DCD rates accounting for up to 50% of all deceased donors [19]. In Austria, the third Eurotransplant member country to implement an active DCD program, DCD accounted for 18% of utilized deceased donors in the year 2024 – a significant increase from 2018, when a controlled DCD (cDCD) program was initiated in the western region of the country. We herein, report on our experience with the initiation of a cDCD program with focus on donor utilization as well as key aspects required for the successfully implementation of such a program.

## Materials and methods

From January 1st, 2018 to December 31st, 2024 all DCD donors referred to the Transplant Center at the Medical University of Innsbruck (MUI) were included in this analysis. Donor data as well as periprocedural data were recorded and analyzed. All included donors corresponded to category III according to modified Maastricht criteria [20].

### Terms and definitions

#### DCD donor

**FIGURE 1:**
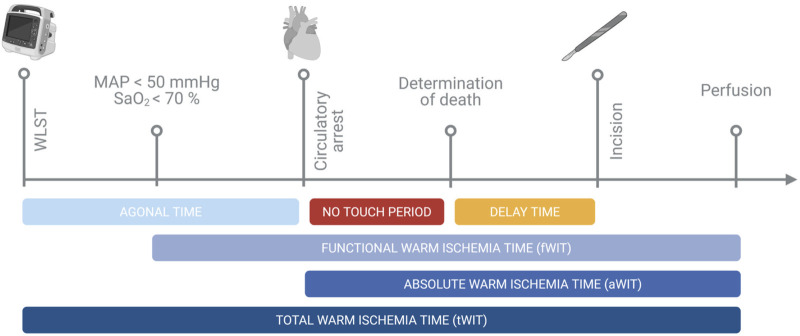
Schematic overview of the controlled DCD pathway and warm ischemia time definitions.

A DCD donor is any donor in whom organ donation occurs after determination of death by circulatory criteria. In clinical practice withdrawal of life-sustaining treatment (WLST) is followed by a variable period of progressive hypoxia and hypotension (functional WIT) until circulatory arrest occurs (Figure 1). In Austria circulatory arrest is determined either through invasive arterial blood pressure measurement or by echocardiography. ECG monitoring is not an effective modality to determine circulatory arrest as electrical activity (i.e., pulseless electrical activity) may still occur despite absence of mechanical activity of the heart.

Circulatory arrest marks the beginning of a mandatory stand-off period, during which no interventions must take place (no-touch period), to ensure no return of spontaneous circulatory activity. The length of the no-touch period differs among countries. In Austria the duration of the no-touch period is 10 min, after which death is confirmed by a standardized clinical examination [19]. This is performed by two fully licensed physicians who are independent of the donation process following a two-person verification principle. In the planning of a DCD procedure, it should be ensured that two physicians are present and available for the determination of death.

#### Potential, referred, actual and utilized DCD donor

Any patient potentially suitable for cDCD is termed a potential donor. Any potential donor that is reported to the organ procuring transplant center following initial screening for medical and legal contraindications is defined as a referred donor. An actual donor is a donor in which the procurement operation is started, while a utilized donor is a donor from whom at least one organ has been transplanted [21].

#### Warm ischemia time (WIT)

Total WIT (tWIT) is defined as the time from WLST to initiation of perfusion. tWIT can be further divided into agonal WIT, functional WIT and absolute (asystolic) WIT. Agonal WIT is the time from WLST to circulatory arrest. Absolute or asystolic WIT is the time from circulatory arrest to the start of perfusion (Figure 1) [22, 23]. The definition of donor WIT (dWIT) or functional WIT (fWIT) varies, even amongst centers within the same country. We defined fWIT as the time-point when the mean arterial pressure (MAP) decreases below 50 mmHg or oxygen saturation (SaO_2_) falls below 70% until the start of perfusion.

### Legal and ethical framework

In Austria organ donation including DCD is regulated by national law. Any critical care physician, who is treating a suitable potential donor is authorized, encouraged and even mandated to report the potential donor–DBD or DCD–to the regional transplant center. However, no standardized referral criteria exist, and referral is left at the discretion of the primary care team. Austria has an op-out system in place where the potential donors would have needed to opt-out during their lifetime to avoid becoming a donor. Thus, for every potential donor, the national objection registry is consulted prior to initiating the formal donor evaluation process. Even though from a legal standpoint organ donation may proceed in the absence of a registered objection, the family is always approached for consent and may also stop the DCD process at any time. Families are approached by the primary care team of the ICU and may be supported by the local transplant coordinator if needed.

Alongside the legal framework, ethical considerations remain central to the DCD process, particularly with regard to antemortem interventions, the separation of end-of-life care from transplant decision-making as well as the maintenance of transparency and public trust [24].

#### Antemortem interventions

There is consensus in Austria that antemortem interventions are only allowed under two conditions:

1. The comfort of the patient is not compromised.
2. The intervention or diagnostic measure serves to improve the transplantation outcome.

The following interventions can currently be performed under these two perspectives:

- Imaging procedures, including advanced imaging such as CT scans
- Bronchoscopy/transesophageal echocardiography under adequate analgesia and sedation
- Premortem administration of heparin
- Premortem administration of cortisone
- Preparing and draping the patient
- Placement of arterial and venous catheters

### Protocol development and implementation

#### Communication and education as starting point

Clear, effective and open communication and education of the public, medical professionals, support staff and donor families are key elements for a successful DCD program. All involved professional groups (intensive care nurses, intensive care physicians, anesthesia nursing staff, anesthesiologists, operating room nursing and support staff) and hospital management should be briefed and informed about the DCD procedure in advance to the start of the program. Regional transplantation coordinators should organize on-site training or support local authorities in conducting such events. Further to this, briefings as well as de-briefings are essential components of an effective communication strategy, ensuring that all stakeholders are aligned. When a DCD procedure is planned, sufficient time for an on-site briefing for the involved physicians, nursing team and operating room support staff should be planned at the donor hospital. During the briefing of the procurement teams, tasks must be clearly defined and assigned. This includes who will be responsible for diagnosing and confirming death (two-person verification principle), who will accompany the relatives of the donor during their stay in the hospital (family discussion, guiding them to the operating room, accompanying them back to the intensive care unit, et cetera), who will inform the retrieval team about the ongoing process and expected end time of the no-touch period as well as briefing of the entire team involved in the organ procurement (e.g., surgeons, nurses, perfusionists, anesthetists, etc.).

Even individuals who are only peripherally involved with the presumptive organ donor, such as the patient porters, should not be overlooked in the information dissemination process.

Following the DCD procedure, a de-briefing should take place with all persons of the donor hospital who were involved in the DCD process, in order to address any potential questions, clarify misunderstandings, and identify potential issues for further improvement. The primary care team of the patient bears the responsibility for the communication process with the donor’s relatives. If necessary, the regional or in-house transplantation coordinator can support the primary care team. Continuity of care and communication before, during and after the donation process is helpful and highly appreciated by the relatives. Before a planned DCD procedure, important aspects that differ from DBD donation should be discussed with the patient’s relatives. These include the following points:

- Explaining the concept of changing treatment goals, palliative analgosedation, WLST and the dying process
- Explaining the DCD procedure
- Clarifying whether the relatives wish to be present during the process of dying
- Explaining that in exceptional cases, if the dying process takes too long, organ donation may not be feasible due to the unavoidable ischemic damage of the organs
- Explaining that investigations are carried out beforehand to assess the suitability of the organs for donation, but ultimately, it can only be determined after organ retrieval whether transplantation is possible
- Introducing the rules of conduct in the operating room (e.g., which parts of the patient may be touched)
- Clarifying if there is a wish for psychological or spiritual support of the family and if the relatives wish another farewell after organ donation, and if so, organization of the appropriate facilities

Additional questions from relatives may arise during the process of dying. These may concern medication administration or monitoring. A member of the primary care team should conduct another meeting with the relatives after organ donation and offer further support if needed. The discussion regarding a possible objection to organ donation by the potential organ donor is conducted after informing the relatives about the unfavorable prognosis of the patient and the planned WLST.

#### Cooperation and coordination between donor hospital and organ procuring transplant center

Once a potential donor is identified, early contact with the procuring transplant center is recommended, and the following topics should be discussed in advance:

- Required medical examinations of the potential donor
- Evaluation of the assumed quality of the potential retrieved organs
- Modality and extent of life-sustaining therapies
- Planned location for WLST (ICU vs. OR)
- Planned presence of relatives of the potential donor at the time of WLST
- Necessity for social or psychological support for the family of the potential donor
- Requirements for potential machine perfusion of the retrieved organs in the OR
- Timing and dosing of cortisone and heparin administration
- Necessity of tracheal re-intubation after determination of death

#### Documentation

The transplant center requires the documentation and transmission of specific time points during the DCD procedure (WLST, desaturation below 70%, drop of blood pressure, cessation of circulation, etc.) which need to be passed on to Eurotransplant and which are also important for the hospital’s internal documentation. The DCD protocol remains in the patient’s medical record. A copy should be provided to the organ procurement team. Until the patient died in the OR, either the ICU documentation is continued, or an anesthesia documentation protocol must be generated. A documentation of the operation is required for the retrieval procedure with a final sign off on the protocol by the retrieval surgeons after final on-site organ assessment and referral of the documentation to the transplant center.

### Clinical pathway for controlled DCD

#### Planning and preparation

Upon identifying a patient as a potential donor, the donor hospital in cooperation with the local transplant coordinator contacts the regional transplant center. The transplant center evaluates the suitability of the potential donor for organ donation based on the available medical findings and by consulting the national registry to exclude any objection against organ donation. Once medical and legal contraindications to organ donation have been excluded, the potential donor is considered a referred donor. The ICU team at the donor hospital together with the transplant coordinators of the regional transplant center–which also deploys the procurement team–then determines a suitable time for WLST and the procurement procedure. At the donor hospital an internal briefing is held as described above.

Once the organ procurement team arrives at the donor hospital an external briefing is convened. The external briefing should cover topics such as donor identification as well as a detailed discussion of the whole donation process to make sure everyone is informed about the individual tasks. Furthermore, maximum tolerable ischemia times specific for each organ are determined based on the donor’s risk profile (pre-existing conditions, age, current organ function, etc.). Throughout the donation procedure a person who is not member of the procurement team, with knowledge and understanding of DCD procedures should be present.

#### WLST and determination of death

The decision to change treatment goals to palliative care and WLST is made, documented and implemented by the primary care team independent from any considerations of organ donation. This decision is carried out according to local medical and ethical protocols. Once the decision has been made, treatment becomes symptom-oriented (e.g., analgesia and sedation) aiming to ensure best supportive care until death occurs. From the time of the decision to change treatment goals until WLST, measures to maintain organ quality are often necessary (circulatory support, volume resuscitation, anticoagulation, potential donor preparation for surgery, etc.). The location of WLST may vary between the donor hospitals, depending on the local conditions. WLST can occur either in the ICU or in the OR. However, it has been proven to be advantageous to withdraw life-sustaining therapy in the OR. In any case, efforts should be made to ensure the shortest possible WIT. Once the patient has been transferred to the OR premortem interventions such as administration of heparin and cortisone as well as prepping and draping of the patient are carried out. Before WLST a modified Team Time Out is performed. The organ procurement team is then asked to step outside the OR and the surgical instruments are covered before WLST commences. The relatives of the patient may be present during the dying process if so desired.

The timepoints of WLST, O_2_-desaturation <70%, and the drop of the MAP ≤50 mmHg, as well as the time of circulatory arrest are documented. The 10-min no-touch period following the onset of circulatory arrest together with the time until organ perfusion is initiated constitutes the asystolic/absolute WIT (Figure 1). Following the no-touch period, death is diagnosed and certified by two physicians using the four-eye principle according to the recommendations of the Austrian Supreme Medical Council. Immediately after determination of death, surgical measures for organ retrieval and preservation are commenced.

#### Surgical procedure

We have implemented a modification of the super-rapid technique first described by the Pittsburgh group in 1995 [25], which has become the standard for abdominal DCD organ retrieval. The anterior abdominal wall is elevated with clamps, and a midline laparotomy from the xyphoid process down to the symphysis pubis using a scalpel is performed. The cut is deepened down to the linea alba and preperitoneal fat. The peritoneal cavity is entered bluntly just above the umbilicus. The small bowel is placed in a lab pad and retracted towards the donor’s left shoulder. The right iliac common artery or distal aorta is incised and cannulated. The aorta is either cross-clamped right below the diaphragm or intrathoracically via a median sternotomy, and the inferior vena cava (IVC) is vented intrapericardially. The abdominal viscera are cooled with sterile ice until perfusion with cold preservation solution is completed.

For heart donation from a cDCD donor, the cardiac surgical team prepares two separate instrument tables to ensure procedural independence from the abdominal retrieval team and the primary scrub nurse. At our institution, a direct procurement and perfusion strategy utilizing normothermic *ex situ* heart perfusion (ESHP) is currently employed. Sharp dissection through the skin and subcutaneous tissue down to the sternum and a median sternotomy are performed. The pericardium is widely opened. A venous cannula is then inserted into the right atrium to collect blood for priming of the ESHP circuit. To ensure adequate priming volume, typically at least 1.2 L, the donor is placed in Trendelenburg position to optimize venous return. Subsequently, a perfusion cannula is inserted into the ascending aorta, followed by intrathoracic aortic cross-clamping. Cold cardioplegic solution is administered into the aortic root and cold saline is intermittently poured over the epicardial surface to maintain topical cooling. From this point onward, the surgical steps mirror those of standard DBD procurement, including myocardial cooling, cardiectomy, and back-table preparation of the graft for *ex situ* perfusion and subsequent transport.

For lung donation from a DCD donor, organ removal is performed in close temporal conjunction with super-rapid abdominal and heart removal, with priority given to minimizing warm ischemia. Following median sternotomy and further opening of the pericardium, the pulmonary trunk is then exposed and cannulated. The left atrium is opened or vented via the left atrial appendage region to relieve the heart. After intrathoracic aortic clamping, a cold perfusion solution in combination with Prostaglandin E (e.g., 250 μg per 3000 mL bag) is applied antegrade via the pulmonary artery, while the lungs are cooled topically with sterile ice or slush. Controlled ventilation is maintained to prevent atelectasis. After complete perfusion and sufficient cooling, the following steps mimic standard DBD procurement steps. Depending on the protocol, back-table procedure includes retrograde irrigation via the pulmonary veins and preparation for transport or subsequent *ex-situ* lung perfusion.

#### Ischemia times and aborted donation

If the agonal WIT (Figure 1) exceeds 120 min the cardiothoracic team stands down, while the abdominal retrieval team will wait a total of 240 min before standing down. Typically, the duration of the agonal phase is significantly shorter. To estimate the expected agonal WIT, the Wisconsin Score can be used [26]. Should a prolonged agonal WIT preclude organ retrieval, at least one member of the treatment team (along with the family) remains in the operating room and awaits the patient’s passing. If, unexpectedly, the dying process extends over several hours, a return to the intensive care unit may be considered. If the fWIT exceeds 60 min, the liver and pancreas are not retrieved. Kidney and lung retrieval is aborted if the fWIT surpasses 120 min. In heart transplantation a fWIT longer than 30 min, precludes organ utilization.

Members of the transplant centers, as well as of the donor hospitals need to be aware that there is a possibility that the dying process may last longer than it is acceptable for organ donation or that other reasons may prevent organ retrieval. This, however, should not deter intensive care physicians from reporting potential organ donors.

## Results

### Donors

A total of 56 Maastricht category III potential donors were referred during the study period. The median donor age was 57 years (range 15–76), 44 donors were male (78.6.%) and 12 female (21.4%).

Of the 56 referred donors, 53 (94.6%) became actual donors. In one referred donor a renal cell carcinoma was suspected based on further workup which ultimately precluded donation. As for the two other cases, in one case the referred donor did not progress to circulatory arrest and was transferred back to the ICU, while in the other case, no suitable recipient could be identified within the Eurotransplant region.

Overall, in 42 cases, cDCD donation resulted in successful transplantation of at least one organ corresponding to a utilization rate of 75.0%. Table 1 shows donor characteristics according to potential, actual, non-utilized and utilized donors. Utilized donors had lower BMIs compared to non-utilized donors (25 kg/m^2^, IQR 23–28 vs. 31 kg/m^2^, IQR 27–32; p = 0.003).

**TABLE 1:** Donor characteristics.

Donor factors	Referred (n = 56)	Actual (n = 53)	Non-utilized (n = 11)	Utilized (n = 42)	*P*-value
Age (years)	57 (50–65)	58 (51–65)	60 (52–69)	57 (49–65)	0.278
Sex	​	​	​	​	0.697
- Female	12 (21.4)	12 (22.6)	3 (27.3)	9 (21.4)	​
- Male	44 (78.6)	41 (77.4)	8 (72.7)	33 (78.6)	​
Blood group	​	​	​	​	0.664
- 0	19 (33.9)	19 (35.8)	5 (45.5)	14 (33.3)	​
- A	23 (41.1)	22 (41.5)	5 (45.5)	17 (40.5)	​
- B	13 (23.2)	11 (20.8)	1 (9.1)	10 (23.8)	​
- AB	1 (1.8)	1 (1.9)	0	1 (2.4)	​
BMI (kg/m^2^)	26 (24–30)	26 (24–29)	31 (27–32)	25 (23–28)	**0.003**
ECMO	​	3 (5.7)	​	​	​
ICU stay (days)	5 (3–7)	5 (3–7)	4 (3–7)	5 (4–7)	0.019
Warm ischemia time (minutes)					
- tWIT	​	27 (24–30)	23 (20–29)	27 (25–30)	0.271
- Agonal WIT	​	12 (9–15)	10 (9–13)	13 (10–15)	0.155
- Asystolic WIT	​	14 (12–15)	14 (13–16)	14 (12–15)	0.664
- fWIT	​	25 (21–28)	22 (19–28)	26 (23–28)	0.363
Time to cannulation	​	3 (2–5)	4 (3–6)	3 (2–5)	0.368

Overview over referred, actual, non-utilized and utilized donor characteristics. A higher donor BMI was associated non-utilization. (31 kg/m^2^ vs. 25 kg/m^2^, p = 0.003). Significant P-values are shown in bold.

For utilized donors the median agonal, absolute and functional WITs were 13 min (IQR 10–15), 14 min (IQR 12–15) and 26 (IQR 23–28) minutes respectively. The median time to cannulation (time from skin incision to start of cold perfusion) was 3 min (IQR 2–5, range). Four donors (7.1%) were on ECMO before WLST. Normothermic regional perfusion (NRP) was employed in one case during the seventh and final year of the study period as part of the donation process according to Italian law, which requires a 20-min no-touch period. In this case the fWIT was 66 min. Liver and left kidney of this donor were successfully transplanted.

### Transplanted organs

The mean number of organs transplanted per actual donor was 2.06. Twenty-two donors (41.5%) were single-organ donors, and of those, ten (45.5%) were kidney-only donors. Utilization rates were highest for kidney (60.4%), pancreas (50.0%), lung (47.8%), heart (45.5%) and liver (41.1%).

**FIGURE 2:**
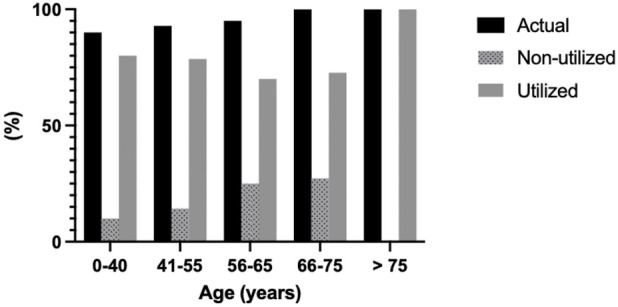
Utilization of controlled DCD donors by age group.

Table 2 provides an overview of all reported, procured and transplanted organs. Figure 2 depicts numbers and percentages of potential, actual, non-utilized and utilized donors according to age groups.

**TABLE 2:** Offered, procured and transplanted organs.

Donor status	Number of donors, n and (%)
Referred donors	56 (100)
Actual donors	53 (94.6)
Utilized donors	42 (75.0)

Organ disposition	Heart	Left lung	Right lung	Liver	Pancreas	Left kidney	Right kidney	Intestine
Offered	11	23	23	56	6	48	48	2
Procured	5	15	15	45	3	39	39	0
Transplanted	5	11	11	23	3	29	27	0
Utilization rate (%)	45.5	47.8	47.8	41.1	50.0	60.4	56.3	0

Values are presented as absolute numbers and percentages in parentheses. Utilization rates were highest for kidneys.

### Influence on donation rates

Following the introduction of the cDCD program in Western Austria, the proportion of DCD donors among all deceased donors in Austria increased from 3% in 2018 to 18% in 2024, despite a concurrent decline in overall deceased donor volume. This decline was largely attributable to the lack of ICU capacities during the COVID-19 pandemic, reflecting global trends observed between 2020 and 2022.

## Discussion

Initiating a DCD program is a multidimensional process requiring (1) the development and implementation of protocols, (2) educating members of the medical community to avoid misconceptions and foster trust and (3) training the medical professionals involved in the DCD procedure. We have outlined the process required to implement and establish such a program in a European setting and highlighted key factors for its success. Furthermore, we report on local protocols and workflows which have been implemented to ensure a standardized pre-, intra- and postprocedural approach.

Implementing a cDCD program has the potential to increase the overall donor volume or at least compensate for static DBD donation rates [27, 28]. A concern that has been voiced is that the implementation of DCD donation programs could negatively impact DBD donation rates [29], which would be undesirable for multiple reasons. One reason is the reduced number of transplanted organs per donor, which, on average is significantly lower for DCD donors compared to DBD donors [30]. Another reason is the additional WIT, which is unavoidably incurred as part of the DCD donation process. Thus, it is important to make sure that implementing a cDCD program does not lead to a shift from DBD to DCD donation but rather an increase in overall deceased donor donation rates. Austrian registry data show overall stable organ donation rates across all regions that have implemented active DCD programs, taking into account an overall decline in organ donation during the COVID-19 pandemic due to a lack of ICU capacities [31]. Since the cDCD program in Innsbruck - covering the Western region of Austria - has been established, the proportion of DCD donation in Austria has increased from 3% to 18%. In the years prior to 2018 there was only a small number of utilized DCD donors in Austria (2017: n = 7, 0.8 PMP; 2016: n = 5, 0.6 PMP; 2015: n = 6, 0.7 PMP; 2014: n = 6, 0.7 PMP; 2013: n = 3, 0.4 PMP.). Almost all these DCD donors were Maastricht category I and were performed by only one Austrian transplant center. Following the implementation of the cDCD program in Innsbruck in 2018, increasing awareness and acceptance of organs from cDCD has resulted in cDCD programs being started in the remaining three Austrian transplant centers covering the Eastern, Central and Southern Region of Austria.

In countries with long-established DCD programs, such as the United States, Spain, the United Kingdom, and the Netherlands, DCD nowadays accounts for up to 50% of all deceased donor organ donation [32]. Steps to further increase DCD activity in Austria and moving towards similarly high rates include continued efforts to raise awareness among all relevant stakeholders, the implementation of nationwide protocols and structured algorithms for the identification and referral of potential DCD donors in ICUs, and the standardization of donor screening processes across the country. In addition, strengthening collaboration between regional transplant centers and local donor hospitals represents an important step toward optimizing the DCD pathway [33]. Additionally, raising public awareness and educating the population on the subject of organ donation and its process through targeted informational initiatives is of crucial importance to foster a supportive environment for organ donation and transplantation.

Key performance metrics in the DCD donation process are the donor conversion (number of actual donors divided by number of referred donors) and utilization rates. In our cohort, the donor conversion and utilization rates were as high as 95% and 75% respectively. An important reason for a referred donor not to become an actual donor is an unacceptably long agonal WIT. The agonal WIT can be highly variable and might be unpredictable. The uncertainty regarding the agonal WIT poses logistical challenges for the donor hospital, the retrieval team as well as the recipient center and may ultimately lead to the abortion of the cDCD procedure which imposes a financial strain and human resource burden on the healthcare system and emotional distress on the donor family and the intensive care team. Thus, different scores and machine learning based models have been developed to predict agonal WIT, identify suitable donors and reduce futile procurements [26, 34–37].

Besides the use of the Wisconsin score [26], an individual evaluation of every potential cDCD donor by an experienced ICU physician of the transplant center allowed us to allocate resources to the donation process only if a high likelihood of donor suitability had been ascertained. This approach has resulted in a median agonal WIT of 12 min (range 1–112 min) and a high donor conversion rate of 94.6%. The consistently high utilization rates across all age categories (Figure 2), along with higher rates in older donors compared to previous studies [21], further underscore the nuanced donor selection process. We consider this a crucial element when establishing a DCD program. High conversion and utilization rates foster trust and confidence in the “new” process among all stakeholders. On the other hand, keeping the selection process too narrow may lead to a significant number of missed referred donors and thus potentially transplantable organs. Therefore, striking a balance between maximizing donor referral and the negative resource and emotional implications of an aborted cDCD procedure outlined above is key–especially in the early phase of implementation.

Current guidelines recommend that cardiothoracic retrieval teams wait for a minimum of two hours following WLST, while for abdominal teams a waiting time of three to four hours is recommended before standing down [38, 39]. These recommendations strike a balance of waiting long enough to not miss out on an actual donor while keeping the logistical aspects somewhat reasonable. The “stand down” process can be further adjusted for individual organs in conjunction with the recipient centers [38]. Within this context, it is important to note that a prolonged agonal WIT does not correlate with an increased fWIT and a prolonged agonal WIT alone is not an independent factor for unfavorable outcomes [40–42]. Rather than agonal or total WIT, fWIT appears to be the key determining factor for posttransplant outcomes in DCD organ transplantation [9].

Unfortunately, fWIT is not uniformly defined complicating comparisons across different countries and sometimes even across different centers within the same country [22, 23, 43]. Thus, an international consensus on fWIT would be desirable. Furthermore, acceptable fWIT times differ for different countries as well as different organs. For liver grafts most countries - except for Italy, where the no-touch period is 20 min long - define a fWIT cutoff of 30 min [29]. For lung and kidney grafts longer fWIT may be tolerated. In current practice, most lung transplant centers consider a fWIT of 60 min or more as a contraindication to lung transplantation [43]. However, fWIT cutoffs continue to be a matter of controversy, and based on ISHLT DCD registry data, the upper limit of tolerable fWIT for lung grafts may even extend 60 min [42]. The longer tolerable fWIT for lung grafts has been attributed to the local oxygen storage capacity in the alveoli as well as the relatively low metabolic demands compared to that of other organs [44]. For kidney grafts fWIT may be even up to 120 min long [45]. Besides fWIT definitions and cutoffs, graft acceptance and utilization rates also vary significantly across organs and countries. DCD utilization rates as low as 5% have been reported for lung DCD grafts in the US [46], with utilization rates approaching 20% in the current era of DCD transplantation [47].

Eden et al. have shown that for liver grafts DCD utilization rates range from 18.9% to 74.2% [29]. Utilization rates were higher at centers and in countries with active machine perfusion programs [29]. The reason for this observation appears to be twofold: (1) normothermic machine perfusion (NMP) allows for viability assessment of the graft, providing a more objective way to decide which organs qualify for transplantation; (2) hypothermic oxygenated machine perfusion (HOPE) offers the possibility for graft reconditioning, leading to more grafts fulfilling predefined viability criteria. Combining a period of graft reconditioning (during HOPE) with controlled rewarming (COR) followed by viability assessment (during NMP) combines the advantages of both technologies, facilitating increased graft utilization rates without causing increased posttransplant complication rates [48, 49]. Similar observations have been made for other organs. DCD heart transplantation was essentially non-existent until a few years ago and has only become possible through the clinical application of NMP [50, 51]. In the context of lung transplantation, *ex vivo* lung perfusion (EVLP) has the potential to elevate the previously low utilization rates to up to 60% [52]. With the increasing use of *ex-situ* machine perfusion preservation, relying on absolute fWIT cutoffs or other donor factors for risk stratification will probably become obsolete as more objective, standardized ways to assess organs become available. This is expected to lead to an increase in DCD graft utilization.

Historically, half of DCD donors were single-organ donors. In 96% of these single-organ donors the kidneys were the only organs that were procured [38]. In our study, 41.5% of actual donors were single-organ donors, with kidney-only donors accounting for 45% of these cases. The mean number of organs transplanted per actual donor was 2.06, consistent with previously reported reference ranges (0.9–2.1) [21, 30]. While *ex-situ* machine perfusion following organ retrieval can lead to improved utilization rates, the possibility of performing normothermic regional perfusion (NRP) before the retrieval process results in both more organs being recovered per donor and more of the recovered organs being utilized for transplantation [53–55]. Since NRP has the potential to enhance organ quality and thus not only leads to increased utilization rates but also improved posttransplant outcomes approaching those of DBD transplantation [56, 57].

Combining the benefits of NRP and *ex-situ* machine perfusion allows to further expand the safe use of extended criteria DCD donors, previously thought to be unsuitable for DCD, thus increasing the overall DCD donor pool [58].

It seems that the clinical implementation of new technologies will help tap into the underused potential of DCD transplantation and that the number of organs transplanted per DCD donor will increase and approach those of DBD donors.

In summary, we have outlined steps and protocols required to successfully implement a cDCD program. Following the implementation, an increase in the overall cDCD volume in Austria has been observed. A survey amongst European countries has shown that many countries without active DCD programs are planning to establish a cDCD program [19]. This is to be commended as one strategy to expand the donor pool. While we acknowledge that the circumstances and legal frameworks might differ from country to country, we hope that our shared experience can be a helpful resource for others who intend to implement a cDCD program. Technological advances in terms of organ recovery, organ reconditioning as well as organ preservation and viability assessment will ultimately result in an increased DCD donor pool and lead to improved utilization rates without jeopardizing outcomes, unlocking the full potential of DCD. Due to the high-quality evidence now available in favor of NRP [55, 59–61], we are currently in the process of establishing and transitioning to an NRP-DCD program.

## Data Availability

The raw data supporting the conclusions of this article will be made available by the authors, without undue reservation.
